# Guava (*Psidium guajava* L.) Leaf Extract as Bioactive Substances for Anti-Androgen and Antioxidant Activities

**DOI:** 10.3390/plants11243514

**Published:** 2022-12-14

**Authors:** Warintorn Ruksiriwanich, Chiranan Khantham, Anurak Muangsanguan, Yuthana Phimolsiripol, Francisco J. Barba, Korawan Sringarm, Pornchai Rachtanapun, Kittisak Jantanasakulwong, Pensak Jantrawut, Chuda Chittasupho, Romchat Chutoprapat, Korawinwich Boonpisuttinant, Sarana Rose Sommano

**Affiliations:** 1 Department of Pharmaceutical Sciences, Faculty of Pharmacy, Chiang Mai University, Chiang Mai 50200, Thailand; 2 Cluster of Research and Development of Pharmaceutical and Natural Products Innovation for Human or Animal, Chiang Mai University, Chiang Mai 50200, Thailand; 3 Cluster of Agro Bio-Circular-Green Industry, Faculty of Agro-Industry, Chiang Mai University, Chiang Mai 50100, Thailand; 4 School of Agro-Industry, Faculty of Agro-Industry, Chiang Mai University, Chiang Mai 50100, Thailand; 5 Department of Preventive Medicine and Public Health, Food Science, Toxicology and Forensic Medicine, Faculty of Pharmacy, University of Valencia, 46100 Valencia, Spain; 6 Department of Animal and Aquatic Sciences, Faculty of Agriculture, Chiang Mai University, Chiang Mai 50200, Thailand; 7 Department of Pharmaceutics and Industrial Pharmacy, Faculty of Pharmaceutical Sciences, Chulalongkorn University, Bangkok 10300, Thailand; 8 Innovative Natural Products from Thai Wisdoms (INPTW), Faculty of Integrative Medicine, Rajamangala University of Technology Thanyaburi, Pathumthani 12130, Thailand; 9 Department of Plant and Soil Sciences, Faculty of Agriculture, Chiang Mai University, Chiang Mai 50200, Thailand

**Keywords:** androgenetic alopecia, anti-hair loss, dermal papilla, DU-145, guava, hair follicle, hair growth, HFDPC, 5α-reductase, *SRD5A*

## Abstract

Leaves of guava (*Psidium guajava* L.) have been used in Thai folk medicine without any supporting evidence as a traditional herbal remedy for hair loss. Androgenetic alopecia (AGA) is chronic hair loss caused by effects of androgens in those with a genetic predisposition, resulting in hair follicle miniaturization. Our objectives were to provide the mechanistic assessment of guava leaf extract on gene expressions related to the androgen pathway in well-known in vitro models, hair follicle dermal papilla cells (HFDPC), and human prostate cancer cells (DU-145), and to determine its bioactive constituents and antioxidant activities. LC-MS analysis demonstrated that the main components of the ethanolic extract of guava leaves are phenolic substances, specifically catechin, gallic acid, and quercetin, which contribute to its scavenging and metal chelating abilities. The guava leaf extract substantially downregulated *SRD5A1, SRD5A2*, and *SRD5A3* genes in the DU-145 model, suggesting that the extract could minimize hair loss by inhibiting the synthesis of a potent androgen (dihydrotestosterone). *SRD5A* suppression by gallic acid and quercetin was verified. Our study reveals new perspectives on guava leaf extract’s anti-androgen properties. This extract could be developed as alternative products or therapeutic adjuvants for the treatment of AGA and other androgen-related disorders.

## 1. Introduction

Guava (*Psidium guajava* L.) is a well-known tropical tree grown in tropical areas that are widely cultivated for fruit [1]. This plant is used as food and traditional medicine due to its pharmacologic properties. Guava leaf possesses a high content of several bioactive compounds, especially phenolic compounds, which contribute to antioxidant and anti-inflammatory activities [2]. The most potent antioxidant found in guava leaves is known as quercetin [2,3]. The primary traditional uses of guava leaves are for the treatment of gastrointestinal illnesses (diarrhea, stomach pain, gastroenteritis, indigestion, and dysentery) and dermatological problems (skin infection, skin aging, and ulcers) [4]. In Thailand, fresh guava leaves have been traditionally used for hair growth promotion. However, supporting evidence of the guava leaves’ anti-hair loss properties has not been identified and investigated.

A multifactorial hair loss condition called androgenetic alopecia (AGA) is distinguished by a particular pattern of baldness. It is a universal skin condition that affects both men and women [5]. It has been revealed that the severity and prevalence of AGA are lower in the Asian populations than in the Europeans [6]. In addition, the prevalence of AGA among men is higher than in women [7]. The prevalence of AGA in Thailand was approximately 39% and increased with advancing age [8]. Hair loss in males initially develops as a bitemporal recession of the frontal hairline, followed by diffuse hair shedding over the crown. Eventually, it reveals full baldness at the crown with a narrow band of hair in a horseshoe shape on the sides and back of the scalp [9]. In addition, female hair loss involves diffuse hair thinning and reducing hair density in the central part of the scalp with a preserved frontal hairline [10].

The hair cycle is a continuous progression from anagen (growing phase) to catagen (regression phase), then to telogen (resting phase), and returning to anagen. AGA is typified by progressive follicular miniaturization and a shortened anagen phase, resulting in the conversion of terminal hairs to shorter and thinner vellus hairs [11,12]. Hair follicular miniaturization is caused by androgens in androgen-sensitive areas of the scalp [13,14]. Furthermore, it has been established that AGA-scalp skin is extremely sensitive to androgen, with androgen production and androgen receptor response significantly increasing in these areas, particularly in hair follicle dermal papilla cells (HFDPC) [6]. Enzyme 5α-reductases convert testosterone into the most potent androgen, dihydrotestosterone (DHT) [15]. Androgen receptor (AR) affinity of DHT is roughly five times higher than that of testosterone [16]. Then, AR-bound DHT promotes hair follicle regression by upregulating the molecules that inhibit hair growth, such as dikkopf-related protein 1 (DKK-1), interleukin-6 (IL-6), and transforming growth factor (TGF-β) [2,17].

There are three isotypes of the enzyme 5α-reductase: type 1, 2, and 3, which are encoded separately by *SRD5A1, SRD5A2*, and *SRD5A3,* respectively [18,19]. Different *SRD5A* gene expression patterns have been seen in androgen-responsive tissues such as the prostate and skin [18]. DU-145 prostate cancer cells (DU-145) express all forms of the *SRD5A* genes. These cells have been used as an in vitro model to examine how herbal extracts or other compounds influence the expression of the *SRD5A* gene in order to screen for antagonism to androgens for the prevention of hair loss. [20,21]. Likewise, HFDPC, which are specialized mesenchymal cells found at the base of hair follicles and are important in hair follicle formation and postnatal hair growth cycles, are used as an in vitro model to assess the cellular and molecular effects of hair growth-moderating substances as well as their effects on 5α-reductase activity [22].

To date, the main treatment options for AGA are synthetic medicines (topical minoxidil and oral finasteride), whereas other alternatives have not yet been approved [23]. The use of finasteride, a competitive inhibitor of 5α-reductases, is restricted in men. However, minoxidil is a common medicine for treating AGA in both genders, which acts through multiple pathways [24]. Side effects of these medications include testicular soreness, erectile dysfunction, skin sensitivity, and scalp dryness [25]. Adherence to hair loss treatments of some individuals with AGA is reduced by these constraints. Since AGA is progressive hair loss and requires long-term treatment, alternative therapies and natural herbal medicines have gained interest because of their benefits, which include a variety of hair growth-promoting actions and affordable prices [26].

The underlying mechanisms of guava leaf extract and its bioactive compounds on the androgen pathway have not been established. We hypothesized that the hair-growth promoting activity of guava leaf may involve androgen production, which is the predominant pathway in AGA pathogenesis. Therefore, the objectives of this study were to assess the effects of guava leaf extract on the expression of genes involved in the androgen pathway in two well-known in vitro models, DU-145 and HFDPC, as well as to identify the bioactive components and antioxidant properties of the extract.

## 2. Results

### 2.1. Extraction Yield and Bioactive Compound Estimation

The extraction yield of guava leaf extract was 19.89 ± 0.20%. The physical appearance of the ethanolic extract after concentration was a viscous semisolid texture with a dark green color. The total phenolic and flavonoid contents of the extract were 117.21 ± 2.05 mg GAE/g and 128.10 ± 1.39 mg EGCGE/g, respectively. In the extract, minor amounts of polysaccharide and protein were detected at roughly 0.29 ± 0.10 mg D-glucose/g and 1.33 ± 0.05 mg BSAE/g.

### 2.2. Characterization of the Phytochemical Profile by Liquid Chromatography–Mass Spectrometry (LC–MS) Analysis

**Figure 1:**
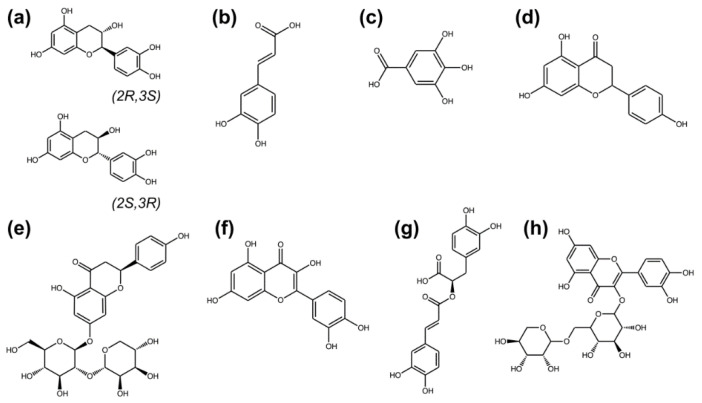
Chemical structures of phenolic compounds in the guava leaf extract that were detected by liquid chromatography–mass spectrometry: (**a**) catechin comprising of (+)-catechin (2R,3S) and (−)-catechin (2S,3R); (**b**) caffeic acid; (**c**) gallic acid; (**d**) naringenin; (**e**) naringin; (**f**) quercetin; (**g**) rosmarinic acid; (**h**) rutin. All chemical structures were acquired from PubChem database [27] and visualized by ChemDraw (version 22, PerkinElmer Informatics).

In Table 1, the amounts of various phenolic compounds found in guava leaf extract are shown, which were detected by LC-MS. Catechin was the most abundant bioactive component (2.215 ± 0.031 mg/g extract), followed by gallic acid (0.751 ± 0.008 mg/g extract), and quercetin (0.520 ± 0.022 mg/g extract). The stereoisomers of catechin, (+)-catechin (2R,3S) and (−)-catechin (2S,3R), were jointly interpreted in this study. Figure 1 illustrates the chemical structures of phenolic compounds in the guava leaf extract.

### 2.3. Antioxidant Activities of Guava Leaf Extract

The scavenging capacities of the extract were estimated by DPPH and ABTS radical scavenging analyses, whereas the metal chelation was evaluated by the ferrous ion chelating assay. The DPPH test demonstrated a similar result (DPPH 444.05 ± 1.01 mg TE/g extract) to that obtained using the ABTS assay (424.80 ± 31.05 mg TE/g extract). Additionally, the guava leaf extract’s capacity to chelate transition metal irons was equivalent to 13.59 ± 0.01 mg of EDTA (the common chelator) per gram of extract.

### 2.4. Effect of Guava Leaf Extract on the Expression of Gene Asscociated with Androgen-Dependent Pathway

According to the cell viability assay, the highest concentration that provided cell viability above 80% in both HFDPC and DU-145 was 62.50 μg/mL (Supplementary Material Figure S1). The following concentrations at 31.25, 15.63, and 7.81 μg/mL were selected to investigate the dose-dependent effects of the guava leaf extract.

We assessed the regulatory impact of guava leaf extract and three main phenolic compounds (quercetin, gallic acid, and catechin) at various concentrations (62.50, 31.25, 15.63, and 7.81 μg/mL) on the mRNA expressions of genes encoding 5α-reductases, including *SRD5A1, SRD5A2*, and *SRD5A3.* Since the most common catechin isomer in plants is (+)-catechin [28], this stereoisomer was chosen for the experiment. Dutasteride, finasteride, and minoxidil were utilized as reference standard substances at the same concentration throughout all experiments. Results from in vitro models of the HFDPC and DU-145 were compared.

**Figure 2:**
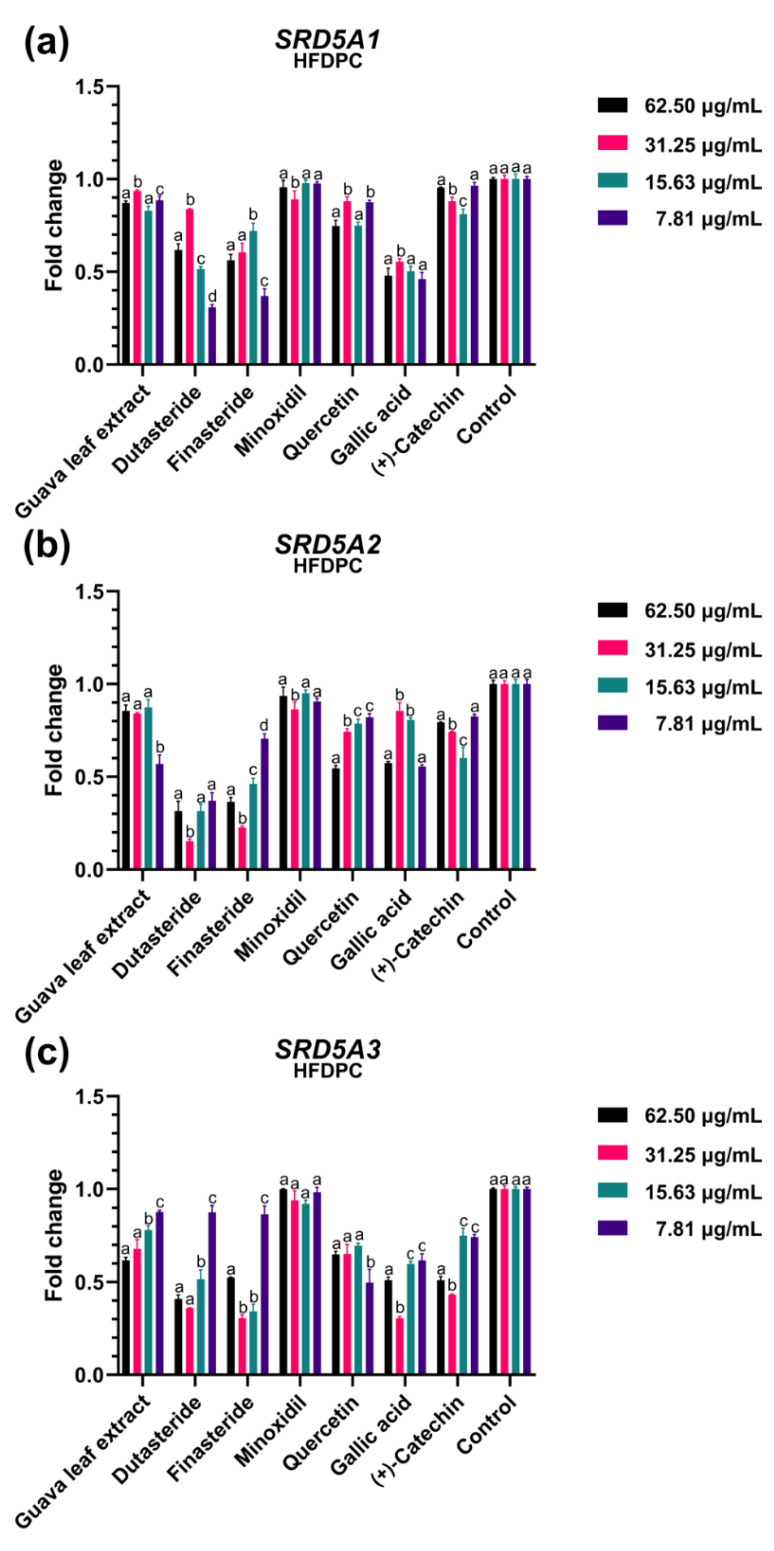
The effects of the guava leaf extract and standard reference compounds (dutasteride, finasteride, minoxidil, quercetin, gallic acid, and (+)-catechin) at different concentrations (62.50, 31.25, 15.63, and 7.81 μg/mL) on the expression of (**a**) *SRD5A1*, (**b**) *SRD5A2*, and (**c**) *SRD5A3* in human hair follicle dermal papilla cells (HFDPC). Values not sharing the same superscript letter are significantly different (*p* < 0.05) when compared between concentrations of each treatment.

The effect of guava leaf extract on *SRD5A* gene expression in HFDPC is depicted in Figure 2. The expression of *SRD5A1* (Figure 2a) and *SRD5A2* (Figure 2b) was marginally downregulated by all concentrations examined, with a fold change range of roughly 0.83–0.94. However, the guava leaf extract at concentrations of 7.81 μg/mL significantly downregulated *SRD5A2* when compared to other concentrations (*p* < 0.05). Additionally, the extract was found to downregulate *SRD5A3* in a concentration-dependent manner (Figure 2c). Among *SRD5A* genes, both dutasteride and finasteride substantially suppressed *SRD5A2* expression. The expression of *SRD5A* in HFDPC was not altered by minoxidil. In comparison to quercetin and (+)-catechin, all concentrations of gallic acid, in particular 62.50 g/mL, considerably downregulated all types of *SRD5A.*

**Figure 3:**
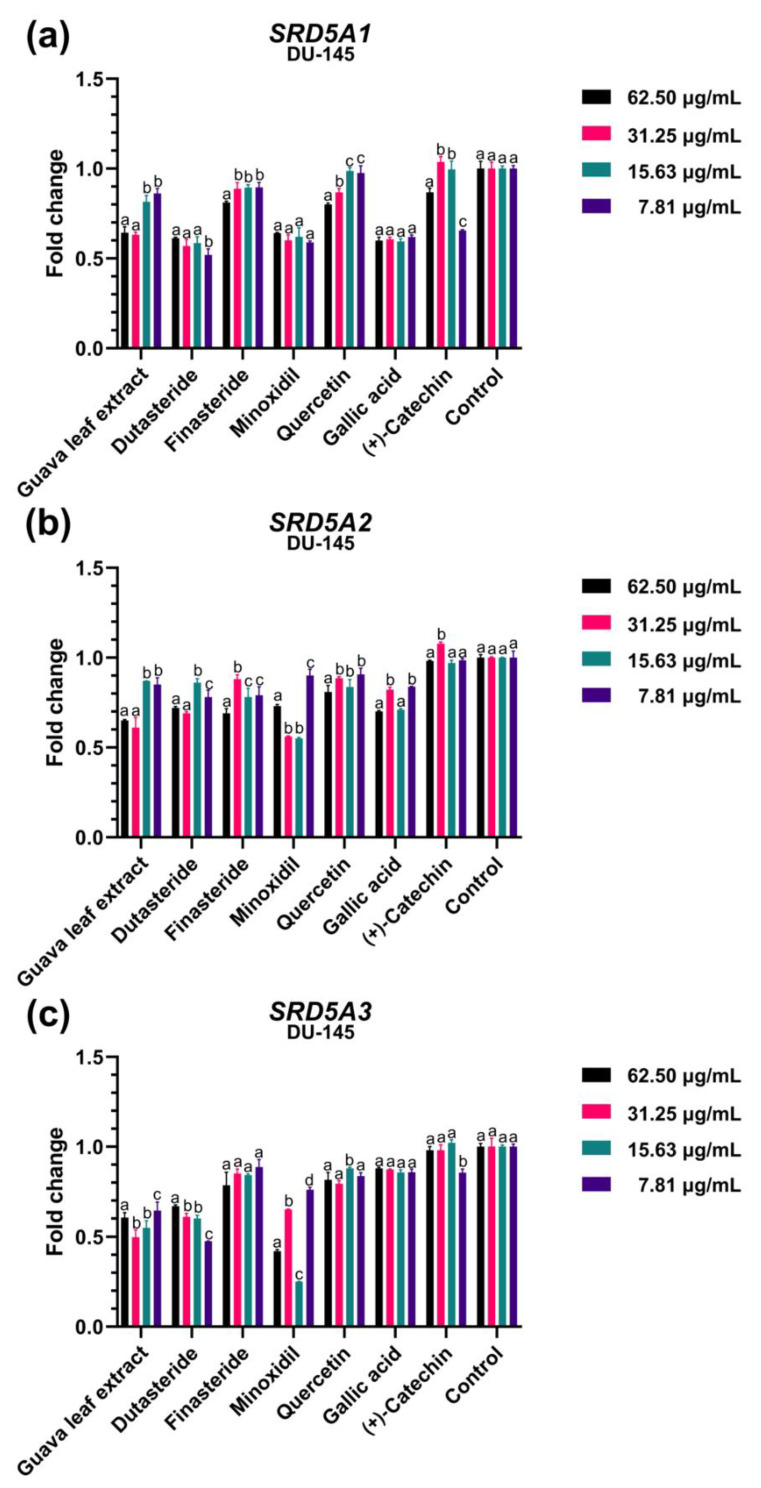
The effects of the guava leaf extract and standard reference compounds (dutasteride, finasteride, minoxidil, quercetin, gallic acid, and (+)-catechin) at different concentrations (62.50, 31.25, 15.63, and 7.81 μg/mL) on the expression of (**a**) *SRD5A1,* (**b**) *SRD5A2*, and (**c**) *SRD5A3* in human prostate cancer cells (DU-145). Values not sharing the same superscript letter are significantly different (*p* < 0.05) when compared between concentrations of each treatment.

Regarding the DU-145 in vitro model, the guava leaf extract at concentrations of 62.50 and 31.25 μg/mL significantly suppressed the expression of *SRD5A1* (Figure 3a) and *SRD5A2* (Figure 3b), when compared to the lower concentrations (*p* < 0.05), with a fold change of about 0.50–0.65. Dutasteride in all examined concentrations appeared to greatly reduce the expression of the *SRD5A1* and *SRD5A3* genes (Figure 3c). Finasteride, however, marginally reduced the expression of all *SRD5A* genes in these cells. Unexpectedly, minoxidil decreased the expression of *SRD5A* genes in DU-145 but not in HFDPC. According to the bioactive compounds, *SRD5A2* and *SRD5A3* expression were modestly attenuated by gallic acid and quercetin. Gallic acid substantially decreased *SRD5A1* expression, whereas the effect of all concentrations was not significantly different. (+)-Catechin had no impact on the expression levels of any *SRD5A* genes in DU-145, which was consistent with the findings of the study using HFDPC.

## 3. Discussion

In Thai folk medicine, *P. guajava* is a significant medicinal plant for hair loss treatment and prevention. Fresh guava leaves are crushed in a mortar, and the resulting paste is then directly applied to the bald spot. Alternatively, fresh leaves are boiled and then the solution is utilized. We conducted this study in order to investigate the guava leaf extract’s beneficial effects on hair loss prevention and to validate its historical use. The finding demonstrated that the guava leaf extract predominantly comprises phenolic components, namely catechin, gallic acid, and quercetin. The scavenging and chelating abilities of the extract were detected. Additionally, the anti-androgenic activity of the guava leaf extract was detected in both the HFDPC and DU-145 models.

Guava leaves are renowned for being an excellent source of phytochemical constituents and present higher amounts of phenolic compounds compared to other vegetable species [29,30]. Likewise, the bioactive estimation in our study verified that the major contents of guava leaf extract were phenolics and flavonoids, with minor presences of polysaccharides and proteins. A previous study reported that the primary phytochemicals of guava leaves are rutin, naringenin, gallic acid, catechins, epicatechins, kaempferol, quercetin, and guaijaverin, which are widely known for their antibacterial, antioxidant, and anti-inflammatory properties [31]. Those compounds were also detected in the extract in this study. Furthermore, the solvent extraction with 50–80% of ethanol has been reported to be an optimum condition for extracting the phenolic compounds in guava leaves [30]. In our study, 70% ethanol was used, from which both the polar and less polar components were co-extracted. Previous study was reported that this condition gave relatively higher antioxidant capacities of guava leaf extract than other extracting conditions [26].

Premature senescence of HFDPC is caused by excessive reactive oxygen species (ROS) accumulation in cells beyond its antioxidative capabilities [32]. Furthermore, metallic impurities, including copper and ferrous sulfate, could be found in hair care products as ingredients or contamination from the manufacturing process [33,34]. The accumulation of these metals in hair follicles accelerates the production of ROS [35]. The radical scavenging and metal chelating abilities of extracts were therefore assessed. A previous study reported that phenolic compounds showed a strong positive relationship with the scavenging activities of the guava leaf extract [36]. Quercetin, rutin, narigin, catechins, caffeic acid, gallic acid, and chlorogenic acid are crucial antioxidative substances in the guava leaves [37,38]. Interestingly, quercetin is recognized as the most powerful and active antioxidant in guava leaves [2]. However, quercetin has a lesser affinity for binding iron than catechin [39]. Our study suggests that the antioxidant properties of the guava extract are synergistically enhanced by the presence of phenolic components, particularly catechin, gallic acid, and quercetin.

In AGA, the shrinkage of hair follicles is mediated by androgens. Balding HFDPC expressed higher levels and activities of AR, 5α-reductases, and DHT than those of non-balding HFDPC [40,41]. Cytoplasmic 5α-reductase enzymes convert testosterone to the potent DHT, which is more potent and has higher affinity towards AR than testosterone [7,42]. The androgen/AR complex binds the androgen response element and recruits transcriptional co-regulators of the target genes [43]. Subsequently, the deregulation of HFDPC-secreted factors, including TGF-β, insulin-like growth factor 1 (IGF-1), WNT family member, and (DKK-1), results in the attenuating proliferation and differentiation of hair follicle stem cells (HFSC) [7].

The protein and mRNA expression patterns of the three isoforms of 5α-reductases differ between species and organs, especially androgen-target organs [19,44]. Remarkably, those enzymes are expressed in both human hair follicles and prostate tissues, which can perform the same reaction to reduce Δ^4^-ene of steroid C-19 and C-21 into a 5α-stereoisomer, producing DHT [19]. The expression of 5α-reductases has been reported to upregulate in AGA scalp [7,45]. Apart from AGA, 5α-reductases play vital roles in the pathogenesis of benign prostate hyperplasia, prostate cancer, and androgen-enhanced skin disorders, including acne vulgaris and hirsutism [46]. Both benign prostatic hyperplasia and AGA can be treated with 5α-reductase inhibitors, such as finasteride and dutasteride [19]. The adverse side effects of 5α-reductase inhibitors have prompted a demand for additional potential inhibitors, particularly those derived from natural sources [26].

Our results show that guava leaf extract and its bioactive components influence *SRD5A* expression in HFDPC and DU-145, especially the latter. Furthermore, the concentration-dependent manner in which the extract suppressed *SRD5A* genes was observed in DU-145 cell lines. Indeed, several studies demonstrated that dutasteride and finasteride diminished the mRNA levels of *SRD5A* genes [47,48,49]. In this study, minoxidil, a potassium channel opener, significantly suppressed the *SRD5A* expression in DU-145, which was consistent with the previous study [50]. In addition, minoxidil was found to be a weak 5α-reductase inhibitor [51]. Gallic acid and quercetin diminished *SRD5A* expression in both HFDPC and DU-145. Our findings were consistent with a prior study that discovered gallic acid decreased the expression of 5α-reductase type 1 and 2 [52]. The ability of quercetin to treat androgen disorders is well known [53]. It possesses antiandrogenic activity by diminishing AR expression and its activity [54]. 

Natural phenolic compounds, including EGCG, catechin, and epicatechin, were found to inhibit 5α-reductase type 1 and 2 isozymes. A catechol group in their structure contributes to the inhibition of 5α-reductase type 1 [55]. However, our result indicated that (+)-catechin did not have a significant impact on the *SRD5A* expression in either HFDPC or DU-145. The (−) form of catechin, however, was not investigated in this study. Additional research is required to determine the proportion of (+) and (−)-catechin in the guava extract and compare the effects of these two stereoisomers on *SRD5A* expression. This study suggests that guava leaf extract might ameliorate androgen-mediated disorders by the effect of bioactive constituents, in particular gallic acid and quercetin, through their anti-androgenic and antioxidant activities.

The differential effects of the extract on decreased *SRD5A* expression in each type of cell may be attributed to the varied amounts of expression of isoforms in HFDPC and DU-145 [18,19]. In addition, it has been discovered that both testosterone and DHT modulate the transcriptional level of *SRD5A* genes in a cell type-specific manner [56,57]. Despite the fact that all *SRD5A* genes have androgen responsive elements in their promoters, androgens control each isoform differently in each tissue [19]. Tissue-specific transcriptional consequences of *SRD5A* genes are most likely influenced by AR co-regulators and transcription factors [19,58]. Additional research is required to determine the effects of chemical substances or the bioactive components in the guava leaf extract on the co-regulators and transcription factors in different tissues.

## 4. Materials and Methods

### 4.1. Chemicals and Reagents

ABTS radical cation (2,2′-azino-bis(3-ethylbenzothiazoline)-6-sulphonic acid), anthrone, DPPH (2,2-Diphenyl-1-picrylhydrazyl, (−)-epigallocatechin gallate (EGCG), gallic acid, sulforhodamine B (SRB), and trolox were obtained from Sigma Chemical (St. Louis, MO, USA). Folin–Ciocalteu reagent was purchased from Merck (Darmstadt, Germany). Dutasteride, finasteride, and minoxidil were from Wuhan W&Z Biotech (Wuhan, China). Agarose gel, Tris base, and 50X Tris/acetic acid/EDTA (TAE) were from Bio-Rad Laboratories (Hercules, CA, USA). Follicle Dermal Papilla Cell Growth Medium Kit (cat no. C-26501) was from Promo Cell GmbH (Heidelberg, Germany). Antibiotic-antimycotic (100X; cat no.1 5240062), fetal bovine serum (FBS; cat no. 16000044), and Roswell Park Memorial Institute medium (RPMI-1640; cat no. 31800022) were from Gibco Life Technologies (Thermo Fisher Scientific, Waltham, MA, USA). Acetic acid, dimethyl sulphoxide (DMSO), trichloroacetic acid, sulfuric acid, and other chemical substances were purchased from RCI Labscan (Bangkok, Thailand). All other chemicals were analytical-grade substances.

### 4.2. Plant Material and Crude Extracts

*P. guajava* leaves were obtained from the Medicinal Plant Garden, Faculty of Pharmacy, Chiang Mai University (Chiang Mai, Thailand) and identified with voucher number PNPRDU63033 at the Pharmaceutical and Natural Products Research and Development Unit, Faculty of Pharmacy, Chiang Mai University. The leaves were dried in a hot air oven at 50 °C for 24 h and ground in a mortar. The powder (40 g) was immersed in 70% ethyl alcohol and boiled in the water bath at 100 °C for 10 min. The supernatant was filtered through Whatman filter paper (Marlborough, MA, USA) and concentrated with low pressure at 50 °C in a rotary evaporator (Hei-VAP, Heidolph, Schwabach, Germany) until the solvent was evaporated, kept in an amber bottle, and stored at −4°C for further analysis.

### 4.3. Bioactive Compound Estimation

#### 4.3.1. Total Phenolic Content (TPC) Assay

The total phenolic content (TPC) was measured by a modified version of a Folin–Ciocalteu colorimetric method [59]. The standard curve was plotted between the measured absorbance and concentration of standard gallic acid. The TPC was calculated using the standard curve equation of gallic acid (y = 13.463x + 0.0406, R^2^ = 0.9991). Results were expressed as milligrams of gallic acid equivalents per gram of extract (mg GAE/g extract). 

#### 4.3.2. Total Flavonoid Content (TFC) Assay

The total flavonoid content (TFC) was estimated by an aluminum chloride colorimetric assay with a slight modification to a previous method [60]. The results were represented as milligrams of EGCG equivalents per gram of extract (mg EGCGE/g extract) through the calibration curve of EGCG (y = 0.3587x + 0.0041, R^2^ = 0.9993).

#### 4.3.3. Total Polysaccharide Content

The sulphuric anthrone was used to quantify the content of carbohydrates in *P. guajava* leaf extract [61]. The content of polysaccharides was calculated based on the established linear curve of D-glucose (y = 43.552x + 0.0765, R^2^ = 0.9996), and represented as milligrams of D-glucose equivalents per gram of extract (mg D-glucose/g extract).

#### 4.3.4. Total Protein Content

Total protein content was determined by the Lowry method [62], adapted to a 96-well plate. Different concentrations of bovine serum albumin solutions were used as a positive control and plotted against its absorbance to create the standard curve (y = 0.0001x + 0.0257, R^2^ = 0.9976). The amount of protein was expressed as milligrams of bovine serum albumin equivalents per gram of extract (mg BSAE/g extract).

### 4.4. Characterization of Psidium guajava Leaf Extract by Liquid Chromatography–Mass Spectrometry (LC–MS)

The extract of *P. guajava* leaves was analyzed using an LC-MS following a method of Arjin et al. [63]. The sample was prepared in 0.01% formic acid and ethanol (1:1, *v/v*) to obtain the final concentration (1 mg/mL), cleaned up in the QuEChERS dispersive SPE kit (Agilent Technology, Santa Clara, CA, USA) to remove fats and pigments, and then filtered through a 0.22 μm pore size membrane filter. Chromatographic analysis was done using an Agilent 1290 Infinity II series (Agilent Tech., Santa Clara, CA, USA), connected with an electrospray ion quadrupole mass spectrometer 6130 (Agilent Tech., Santa Clara, CA, USA). A Restek Ultra C18 reversed-phase column (250 × 4.6 mm, 5 µm, Restek Corporation, Bellefonte, PA, USA) was used to separate the compounds. The injection volume was 5 μL. The gradient elution was scheduled at the following intervals: 80% A at 0–8 min, 80% to 25% A at 8–24 min, 25% A at 24–28 min, 25% to 70% A at 28–34 min, 70% to 80% A at 34–36 min, and 80% A at 36–45 min. The flow rate and column temperature were set at 0.5 mL/min and 30 °C. Mass spectrometry was operated in an electrospray ionization (ESI) probe in negative mode. The nebulization gas was set at 60 psi with nitrogen gas at a flow of 12 L/min. The dry heater temperature was maintained at 350 °C. The capillary voltage was set at –3 kV. A mass scan ranging from 100 to 1200 *m/z* was carried out with an acquisition rate of 250 ms/spectrum. The limit of detection (LOD) and limit of quantification (LOQ) of each standard compound were estimated (Supplementary Material Table S1).

### 4.5. Antioxidant Activities of Psidium guajava Leaf Extract

#### 4.5.1. DPPH Radical Scavenging Assay

The radical scavenging activity of *P. guajava* leaf extract was assessed by DPPH assay with a slight modification to previous methodology [64]. In brief, 50 μL of *P. guajava* leaf extract, standard trolox (0.02–0.4 mg/mL), or blank (distilled water) was added to the corresponding well in a 96-well microplate, followed by the addition of 150 μL of 0.1 mM ethanolic DPPH solution. The mixtures were mixed and left in the dark for 30 min at 25 °C. Then the absorbance of each mixture was measured at 515 nm by a microplate reader (EZ Read 400, Biochrom, Cambridge, UK). The results were expressed as milligrams of trolox equivalents per gram of extract (mg TE/g extract).

#### 4.5.2. ABTS^•+^ Radical Scavenging Assay

The ABTS^•+^ radical scavenging activity of *P. guajava* leaf extract was achieved using the method described by Sridhar et al. [65] with a minor modification. Aqueous ABTS (7mM) was reacted with 2.45 mM aqueous potassium persulphate (1:1, *v*/*v*), and the mixture was left in the dark for 16 h at 25 °C. Then, the ABTS^•+^ solution was diluted with absolute ethanol to obtain a working solution with the absorbance at 730 nm of 0.70 ± 0.20. A total of 40 μL of sample and 160 μL of ABTS^•+^ working solution were added to 96-well plate, then incubated in the dark for 10 min at room temperature. Standard trolox (0.02–0.4 mg/mL) and distilled water were used as a positive control and blank, respectively. Then the absorbance of the mixture was read at 730 nm by a microplate reader (EZ Read 400, Biochrom, Cambridge, UK). The results were expressed as milligrams of trolox equivalents per gram of extract (mg TE/g extract).

#### 4.5.3. Metal Chelating Assay

Metal chelating activity on ferrous ions was measured as described previously [66,67]. Briefly, 50 μL of 2 mM ferric chloride was added to 100 μL of sample and then mixed with 50 μL of 5 mM ferrozine. Standard EDTA (0.02–0.4 mg/mL) and distilled water were used as a positive control and blank, respectively. After 10 min of incubation, all the absorbances were detected at 515 nm by a microplate reader (EZ Read 400, Biochrom, Cambridge, UK). Milligrams of EDTA equivalents per gram of extract (mg EDTAE/g extract) were the measurement unit.

### 4.6. Cell Lines and Culture

Primary human follicle dermal papilla cells (Promo Cell GmbH, Heidelberg, Germany) were grown in the Growth Medium Kit supplemented with 10% FBS and 1% antibiotic-antimycotic 100X solution. DU-145 human prostate cancer cells (American Type Culture Collection, Rockville, MD, USA) were cultured in RPMI-1640 containing 10% FBS and 1% antibiotic-antimycotic 100X solution. Cells were maintained at 37 °C with 5% CO_2_ in a humidified atmosphere. 

### 4.7. Cell Viability Assay

The cytotoxic potential of the *P. guajava* leaf extract was determined by the sulforhodamine B (SRB) assay [68]. Cells were grown in 96-well plates (10^4^ cells/well) for 24 h. The monolayer cells were treated with the extract (0–1000 μg/mL) and standard references (finasteride, dutasteride, minoxidil, (+)-catechin, gallic acid, and quercetin). After 24 h, cultured cells were fixed on plates, washed and dried, and stained with sulforhodamine B solution. Tris-EDTA buffer was used to solubilize the dye extracted from stained cells. The intensity was measured by a microplate reader (EZ Read 400, Biochrom, Cambridge, UK) at 515 nm. The highest concentration providing the percentages of cell viability above 80% was considered as non-cytotoxicity and was selected for further experiments. The percentage of cell viability was calculated by Equation (1), where OD denotes optical density:

(1) $$Cell\;viability\;\left(\%\right)=\left(\frac{OD_{sample}-OD_{blank}}{OD_{control}-OD_{blank}}\right)\times 100$$

### 4.8. RT-PCR Analysis

Total RNA was extracted from cells using the E.Z.N.A.^®^ Total RNA Kit I (Omega Bio-Tek, Georgia, USA). Qubit™ RNA HS Assay Kit and Qubit™ 4 fluorometer (Invitrogen, Carlsbad, USA) were utilized to quantify RNA concentration of samples. Gene expression levels were carried out by the semi-quantitative RT-PCR [69]. Complementary DNA was synthesized using MyTaq™ One-Step RT-PCR Kit (Bioline, Memphis, TN, USA). The following primer sequences were used. *SRD5A1*: AGCCATTGTGCAGTGTATGC and AGCCTCCCCTTGGTATTTTG; *SRD5A2*: TGAATACCCTGATGGGTGG and CAAGCCACCTTGTGGAATC; *SRD5A3*: TCCTTCTTTGCCCAAACATC and TCCTTCTTTGCCCAAACATC; *GAPDH*: GGAAGGTGAAGGTCGGAGTC and CTCAGCCTTGACGGTGCCATG. The RT-PCR products were detected using agarose gel electrophoresis [69]. Gel Doc™ EZ System (Version 3.0; Bio-Rad) and Image Lab™ software (Bio-Rad) were used to estimate band intensity. The relative expression value for the target genes was calculated by normalizing the *GAPDH* expression value. Three duplicates of each sample were examined.

### 4.9. Statistical Analysis

Data were expressed as the mean ± SD of three experiments. Comparisons among groups were analyzed using one-way ANOVA followed by Tukey’s test served in GraphPad Prism version 9.4.0 for MacOS (GraphPad Software, San Diego, CA, USA). Statistical significance was defined as a *p*-value less than 0.05.

## 5. Conclusions

This investigation showed that the guava leaf extract was primarily composed of phenolic compounds, notably catechin, gallic acid, and quercetin, which support its free radical scavenging, chelating, and anti-androgenic properties. Intriguingly, the extract and its bioactive compounds, particularly gallic acid and quercetin, downregulated *SRD5A* genes in both the HFDPC and DU-145 models. Due to the bioactive components in the guava extract acting in a cell type-specific manner, their anti-androgenic activity in DU-145 was more evident than in HFDPC. The results suggest that the guava leaf extract may promote hair growth by reducing free radicals and attenuating the expression of 5α-reductases. This extract may be further developed into alternative products or therapeutic adjuvants for treating AGA as well as other androgen-related disorders.

## Figures and Tables

**Table 1:** Liquid chromatography–mass spectrometry data of compounds detected in *Psidium guajava* leaf extract.

Compound Name	Molecular Formula	*m/z*	Content (mg/g Extract)
Catechin *	C_15_H_14_O_6_	290.27	2.215 ± 0.031
Caffeic acid	C_9_H_8_O_4_	180.16	0.074 ± 0.001
Epicatechin **	C_15_H_14_O_6_	290.26	Nd.
Epigallocatechin gallate	C_22_H_18_O_11_	458.37	Nd.
Gallic acid	C_7_H_6_O_5_	170.20	0.751 ± 0.008
Naringenin	C_15_H_12_O_5_	272.25	0.066 ± 0.001
Naringin	C_27_H_32_O_14_	580.50	0.045 ± 0.001
*p*-Coumaric acid	C_9_H_8_O_3_	164.15	Nd.
Quercetin	C_15_H_10_O_7_	302.23	0.520 ± 0.022
Rosmarinic acid	C_18_H_16_O_8_	360.30	0.039 ± 0.018
Rutin	C_27_H_30_O_16_	610.50	0.109 ± 0.001

* = Mixture of (+)-catechin (2R,3S) and (−)-catechin (2S,3R); ** = Mixture of (−)-epicatechin (2R,3R) and (+) epicatechin-(2S,3S); Nd. = not detected; limit of detection (LOD) of epicatechin = 0.007 mg/g; LOD of epigallocatechin gallate = 0.011 mg/g; LOD of *p*-coumaric acid = 0.007 mg/g.

## Data Availability

Not applicable.

## Supplementary Materials

The following supporting information can be downloaded at: https://www.mdpi.com/article/10.3390/plants11243514/s1, Figure S1. Cell viability of the guava leaf extract by the sulforhodamine B (SRB) assay: (a) hair follicle dermal papilla cells (HFDPC); (b) human prostate cancer cells (DU-145); Table S1. Precision of the method, linearity data for calibration curves, and retention time (RT) of reference phenolic compounds.
