# Supplementary material for: Guava (Psidium guajava L.) Leaf Extract as Bioactive Substances for Anti-Androgen and Antioxidant Activities
Source: Plants (Basel). 2022 Dec 14;11(24):3514. doi: 10.3390/plants11243514 (PMC9784754; doi:10.3390/plants11243514)
Supplement: Supplementary file 1 [file plants-11-03514-s001.zip › plants-2033392-supplementary.docx]

**Figure S1** Cell viability of the guava leaf extract by the sulforhodamine B (SRB) assay: **(a)** hair follicle dermal papilla cells (HFDPC); **(b)** human prostate cancer cells (DU-145)

**(a)**

**(b)**


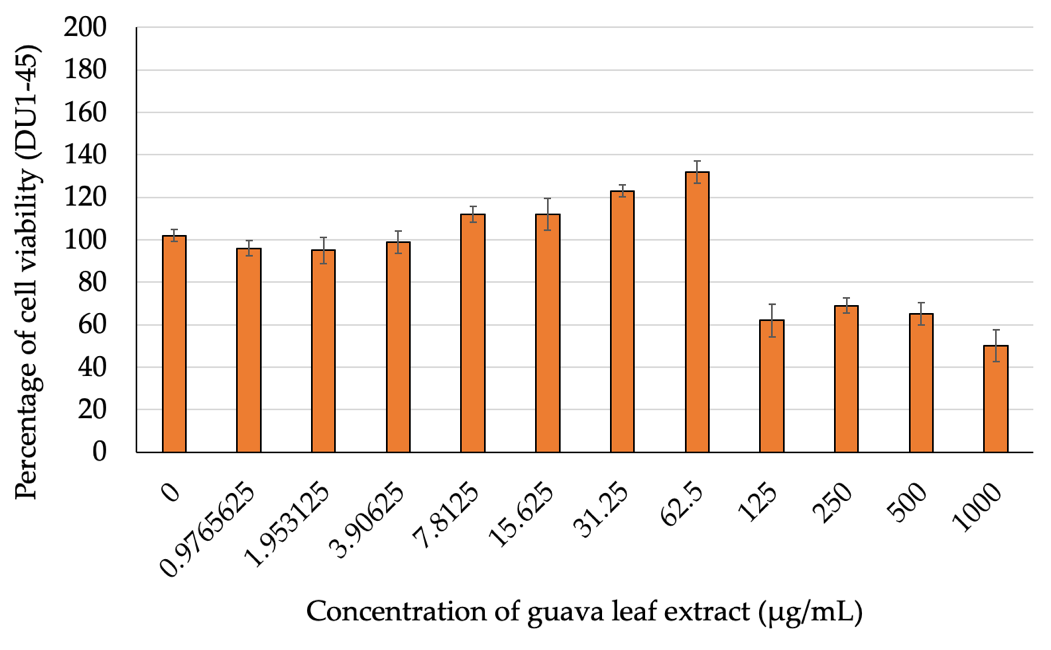

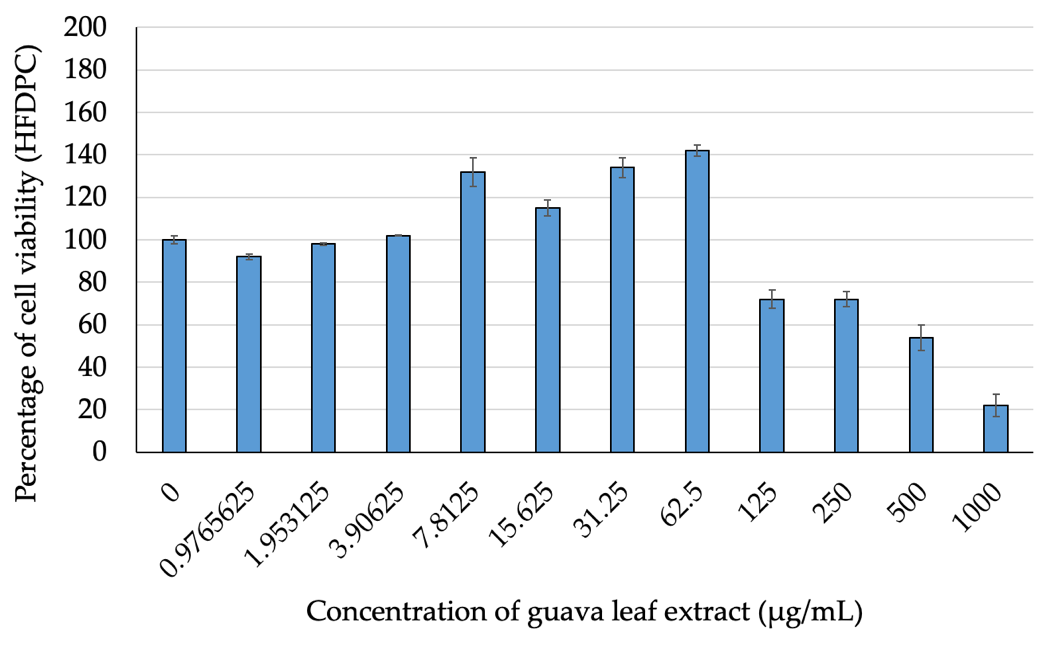


**Table S1** Precision of the method, linearity data for calibration curves and retention time (RT) of reference phenolic compounds

| Compounds | Linearity (μg/ml) | Retention time (min) | Regression equation | R^2^ | LOD (mg/g) | LOQ (mg/g) |
| --- | --- | --- | --- | --- | --- | --- |
| Gallic acid | 1.56-100 | 5.491 | Y=23427x+69424 | 0.9967 | 0.012 | 0.038 |
| Catechin* | 1.56-100 | 7.643 | Y=22288x-26383 | 0.9959 | 0.030 | 0.092 |
| EGCG | 1.56-100 | 8.274 | Y=19629x+30261 | 0.9994 | 0.011 | 0.033 |
| Epicatechin** | 1.56-100 | 9.003 | Y=22893x+35896 | 0.9991 | 0.007 | 0.020 |
| Caffeic acid | 1.56-100 | 10.326 | Y=32304x+158073 | 0.9972 | 0.006 | 0.018 |
| Rutin | 1.56-100 | 14.603 | Y=36167x-29279 | 0.9999 | 0.107 | 0.323 |
| *p*-Coumaric acid | 1.56-100 | 16.579 | Y=13611x+50444 | 0.9966 | 0.007 | 0.022 |
| Naringin | 1.56-100 | 18.848 | Y=32805x+50054 | 0.9987 | 0.014 | 0.045 |
| Rosmarinic acid | 1.56-100 | 20.158 | Y=61663x+161921 | 0.9965 | 0.088 | 0.266 |
| Quercetin | 1.56-50 | 23.378 | Y=65302x+142589 | 0.9943 | 0.378 | 1.147 |
| Naringenin | 1.56-100 | 25.210 | Y=61141x-78948 | 0.9974 | 0.056 | 0.169 |

LOD: limits of detection; LOQ: limits of quantification; *= Mixture of (+)-catechin (2R,3S) and (−)-catechin (2S,3R); **= Mixture of (−)-epicatechin (2R,3R) and (+) epicatechin-(2S,3S);
